# One year results of intravitreal ranibizumab monotherapy for retinal angiomatous proliferation: a comparative analysis based on disease stages

**DOI:** 10.1186/s12886-015-0172-2

**Published:** 2015-12-21

**Authors:** Young Gun Park, Young-Jung Roh

**Affiliations:** Department of Ophthalmology, Yeouido St. Mary’s Hospital, College of Medicine, The Catholic University of Korea, #62 Yeouido-dong, Yeongdeungpo-gu, Seoul 150-713 Korea

**Keywords:** Ranibizumab, Retinal angiomatous proliferation, Stages, Recurrence rate

## Abstract

**Background:**

Retinal angiomatous proliferation (RAP) has been known as a variant of exudative age-related macular degeneration (AMD) with a unfavorable prognosis. To evaluate the effect of ranibizumab administered initially as three loading doses for patients with various stages of RAP.

**Methods:**

A retrospective chart review of 40 patients (41 eyes) with RAP was conducted. The study divided patients into three groups of Group I (8 eyes in stage I), Group II (17eyes in stage II), and Group III (16 eyes in stage III). All patients received three initial monthly intravitreal injections (0.5 mg) of ranibizumab and were monitored monthly for 12 months. Reinjection of ranibizumab after three initial monthly doses was administered on as-needed basis. The main outcome measures were the change in the mean of best-corrected Snellen visual acuity (BCVA) and central macular thickness (CMT), and the total number of injections received during the 12 months.

**Results:**

The mean change in BCVA at 12 months was-0.286,-0.165, and-0.151 (logMAR) in Group I, II, and III, respectively. CMT was also reduced by a mean of 32.72 ± 56.75, 57.45 ± 56.48 and 148.37 ± 98.59 μm. The mean number of injections in Group I was significant lower than those in Group II and III (*P* < 0.001, *P* < 0.001, and *P* = 0.15 for Group I versus Group II, Group I versus Group III, and Group II versus Group III, respectively).

**Conclusions:**

The 12-month follow-up outcomes suggest that three consecutive loading doses of intravitreal ranibizumab is an effective treatment on early stage (stage I) of RAP. Patients in stage I showed a significantly lower recurrence rate than patients in later stages.

## Background

Retinal angiomatous proliferation (RAP) has been described as a variant of exudative age-related macular degeneration (AMD). It was first described by Yannuzzi et al [1] in 2001 and has been differentiated into 3 stages based on clinical and angiographic observations: stage I, intraretinal neovascularization (IRN) with proliferation of intraretinal capillaries within the deep retinal layers, producing intraretinal and superficial retinal hemorrhages; stage II, subretinal neovascularization (SRN). SRN with retinal–retinal anastomosis is further divided into two further categories depending on the absence (IIA) or presence (IIB) of pigment epithelium detachment (PED). Stage III is clinically or angiographically observed choroidal neovascularization (CNV) with vascularized PED and retinal-choroidal anastomosis.

RAP is sometimes referred to as type 3 neovascularization to distinguish it from the Gass CNV type 1 and type 2 anatomic classifications [2]. The natural course of RAP is believed to be worse than that of typical exudative AMD [3]. The risk of neovascularization in the fellow eye is higher in patients with RAP than in those with other forms of neovascular AMD [4]. Different treatment modalities have been proposed for RAP such as conventional laser photocoagulation, transpupillary thermotherapy, surgical ablation, and monotherapy with photodynamic therapy (PDT) using verteporfin (Visudyne; Novartis Pharma AG, Basel, Switzerland), or combined PDT and intravitreal injection of triamcinolone acetonide [5–10].

Although these modalities have been used to treat RAP, their results showed limited success or poor functional outcomes. Vascular endothelial growth factor (VEGF) is well known to be closely related to CNV complexes [11, 12]. Anti-VEGFs including bevacizumab (Avastin®, Genetech, South San Francisco, California) and ranibizumab (Lucentis®, Genetech, South San Francisco, California) have shown promising results in AMD. Major clinical trials have reported that ranibizumab was effective in improving visual acuity (VA) in AMD patients [13, 14]. The demonstration of positive immunoreactivity of VEGF in patients with RAP supports the important role of anti-VEGF agents in treating exudative AMD [15, 16]. Recently, several authors have published the results of treatment of RAP with anti-VEGF monotherapy [17–20]. A different response to treatment in RAP compared with the response shown by typical exudative AMD was reported because of differences in patient’s characteristics, study design and treatment protocol. Although ranibizumab monothrapy did not result in a good response in some reports, it showed promising efficacy and safety for treatment of RAP in other reports. The stage of RAP could be considered to be an important prognostic factor. But few studies have determined the effect of ranibizumab monotherapy based on the RAP stage.

In this study, we report our 1-year results of using intravitreal ranibizumab “loading dose” monotherapy for the treatment of RAP by investigating the change in vision, and foveal height on optical coherence tomography (OCT). To evaluate the efficacy of ranibizumab according to RAP stage, a retrospective subgroup analysis was performed.

## Methods

### Patient selection

We performed a retrospective review of the medical records of all patients who had been treated with intravitreal ranibizumab for three consecutive months for RAP lesions at Yeouido Saint Mary’s Hospital between January 2009 and January 2011. Inclusion criteria was age ≥ 55 years and the presence of a typical RAP lesion, including preretinal, intraretinal and/or subretinal hemorrhages with neovascularization in one of the three stages of RAP confirmed by high definition-optical coherence tomography (HD-OCT), fluorescein angiography (FA) and indocyanine green angiography (ICGA). Forty patients (41 eyes) with RAP were defined with Group I (8 eyes in stage I), Group II (17 eyes in stage II) and Group III (16 eyes in stage III) according to a classification system proposed by Yannuzzi et al. Patients previously treated with PDT or drugs including ranibizumab were excluded from the study. The Institutional Review Board/Ethics Committee of Yeouido St. Mary’s Hospital approved this study (No.SC15RISI0008) and it was performed in accordance with the tenets of the Declaration of Helsinki. All patients provided written informed consent.

### Methods and injection procedure

At baseline, each patient underwent best-corrected Snellen visual acuity testing, slit lamp examination, fundus examination, fundus fluorescein angiography (FFA), indocyanine green angiography (ICGA) using confocal scanning laser ophthalmoscope (Heidelberg Engineering, Heidelberg, Germany), and optical coherent tomography (Cirrus HD-OCT, Carl Zeiss, Dublin, CA, USA). RAP was diagnosed based on preoperative baseline FFA, ICGA and HD-OCT.

All patients received a monthly injection of 0.05 mL ranibizumab for 3 months and were followed up monthly. All follow-up visits included determination of best-corrected visual acuity (BCVA) and examination to measure the central thickness of the neurosensory retina on HD-OCT. FFA and ICGA were performed on month 4 after three monthly loading injections. If any signs indicating relapse were observed on fundoscopy, FFA and ICGA were performed additionally.

In all patients, the main outcome measurements included the changes in BCVA and CMT (as documented by HD-OCT). Central macular thickness (CMT) was defined as the distance between the internal limiting membrane and the RPE. The secondary outcome measurements included persistent leakage on FFA and ICGA. Data on BCVA changes and HD-OCT features were analyzed at the 1-, 2-, 3-, 6-and 12-month follow-up time points. Additional ‘as needed’ injections of ranibizumab were offered at the discretion of the treating physician if a new hemorrhage was observed during a clinical examination or if any signs indicating the recurrence of RAP leakage (intraretinal edema, intraretinal vascular anastomosis, retinal-choroidal anastomosis, or the presence of pigment epithelial detachment) was seen on OCT, FFA or ICGA examination. Subsequent injections were given at least 4 weeks after the previous injection.

### Statistical methods

Snellen visual acuities were converted to the logarithm of the minimum angle of resolution (logMAR) scale for statistical analysis. A decrease in VA was defined as the doubling of the visual angle (≥0.3 on logMAR). An increase in VA was defined as the halving of the visual angle (≤0.3 on logMAR). If the VA change was less than 0.3 on logMAR, it was classified as stable. If the VA changed by a smaller amount, it was classified as stable. The number of treatments performed during the first 12 months after the initial treatment was compared among the groups (One-way ANOVA on ranks). The Wilcoxon signed-rank test was performed to compare any two time points within the group, and an analysis of variance test was used to make a comparison between the groups. *P* values of 0.05 or less were considered to be statistically significant. Statistical analysis was performed using SPSS 17.0 (SPSS, Inc, Chicago, IL).

## Results

The study included 41 eyes from 40 patients with a mean age of 67.09 (SD 11.76) years (range, 46-87). Table 1 shows the main baseline characteristics between the RAP stages. Group III had a significantly lower baseline visual acuity than Group I (*P* < 0.001) and Group II (*P* = 0.030).

**Table 1:** Baseline characteristics of patients with retinal angiomatous proliferation

	Group I	Group II	Group III	*p* value
Mean age (years)	64.75 ± 14.58	63.41 ± 11.04	72.18 ± 9.68	0.180
Number (Male/Female)	8 ( 4/4)	17 (7/10)	16 (5/11)	0.662
Mean BCVA (logMAR)	0.441 ± 0.24	0.602 ± 0.26	0.831 ± 0.56	0.105
Mean CMT (μm)	254.43 ± 57.29	289.78 ± 66.46	372.72 ± 127.14	0.040^*^

BCVA, best-corrected visual acuity; CMT, central macular thickness

^*^ *P* < 0.05

**Fig. 1:**
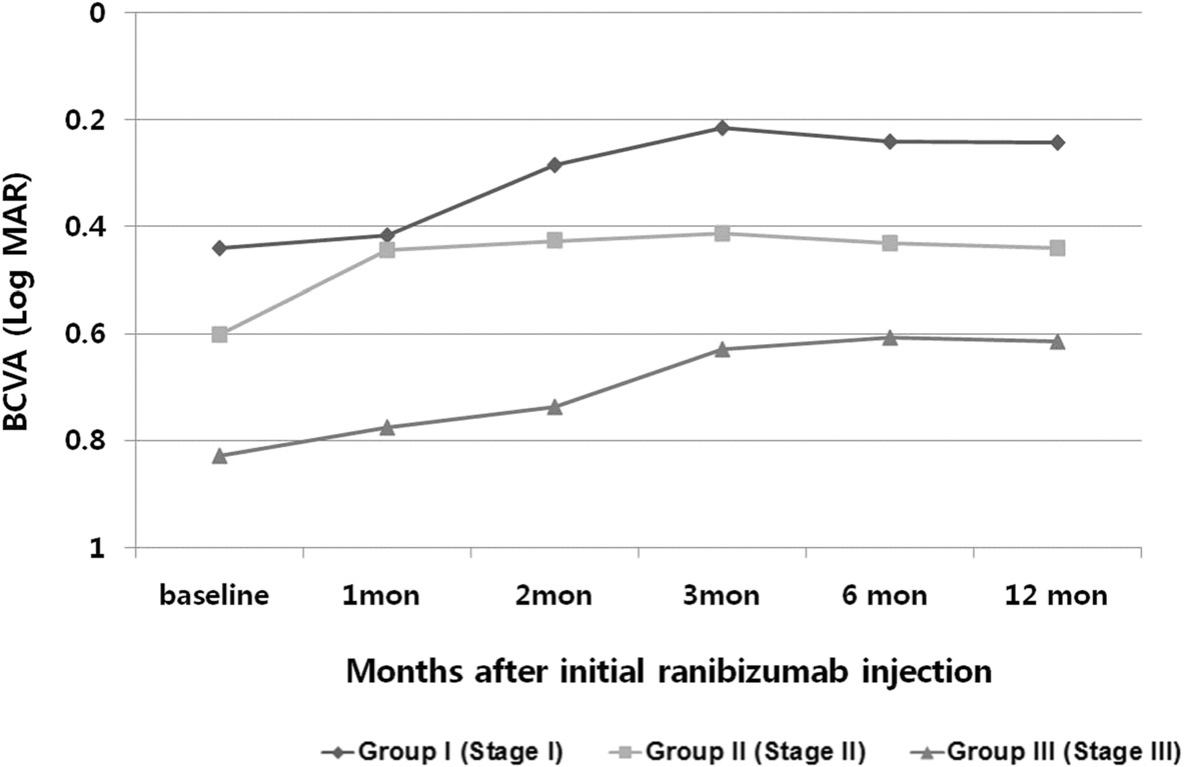
Change in logarithm of the minimum angle of resolution (logMAR) visual acuity over 12 months for 41 eyes treated with an intravitreal injection of ranibizumab according to stage

**Fig. 2:**
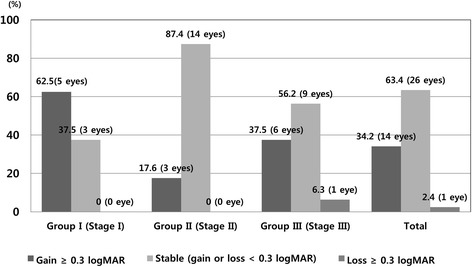
One year results of best-corrective visual acuity (log MAR) change in three groups after ranibizumab injections

BCVA improved from the baseline to the final visit by a mean of-0.29,-0.17,-0.15 log MAR in Group I, II, and III, respectively. The change in mean BCVA at 12 months after initial treatment was better than baseline BCVA in all groups (Fig. 1). After 12 months, forty eyes of 41 eyes (97.5 %) showed improved or maintained BCVA. Only 1 eye in Group III (2.4 %) lost 0.3 logMAR units or more of visual acuity. Five eyes of 8 eyes (62.5 %) in group I showed improvement in BCVA with ≥0.3 logMAR units (Fig. 2).

**Fig. 3:**
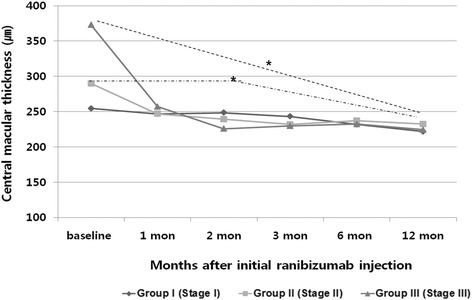
Changes in mean central macular thickness (CMT) of three groups in 12 months

The mean CMT was reduced significantly by 12 months in group II and group III. Although mean CMT in group I was also decreased from 254.43 ± 29.13 μm to 221.71 ± 33.17 μm, the change was not significant (*P* = 0.209). In Group I, II, and III, CMT was reduced by a mean of 32.72 ± 56.75, 57.45 ± 56.48 and 148.37 ± 98.59 μm, respectively. The difference in mean CMT between baseline and the 12-month visit was 24.73 μm between Group I and II (*P* = 0.662), 90.92 μm (*P* < 0.001) between Group II and III, and 115.65 μm (*P* = 0.003) between Group I and III (Fig. 3).

**Fig. 4:**
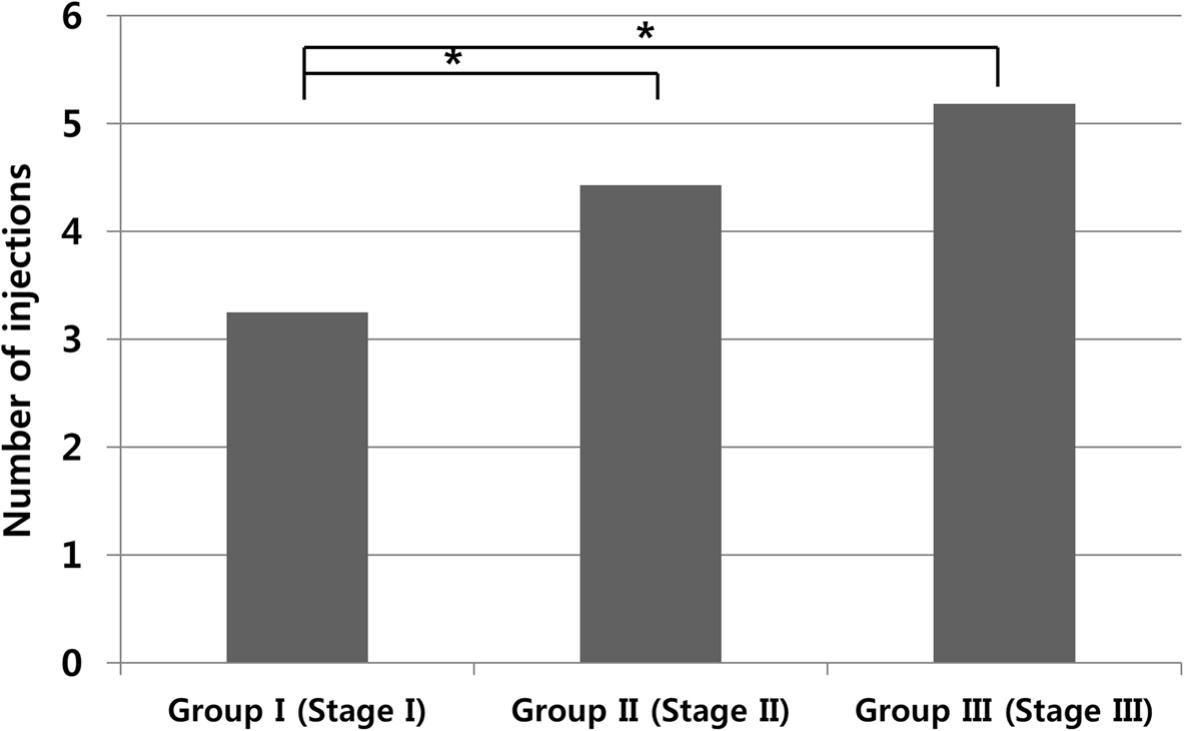
The total numbers of intravitreal ranibizumab injections given during 12 months of follow-up for 41 eyes with retinal angiomatous proliferation (RAP) (* *P* < 0.001)

The mean number of ranibizumab injections received by patients during the 12 months was 4.07. The mean number of injections in Group I, Group II and Group III after initial three consecutive doses of ranibizumab was 0.25, 1.43 and 2.18 respectively. During the 12 months of follow-up after the initial treatment, the mean number of treatments was significantly lower in Group I than in Group II and Group III (one-way ANOVA on ranks: *P* < 0.001, *P* < 0.001, and *P* = 0.15 for Group I versus Group II, Group I versus Group III, and Group II versus Group III, respectively) (Fig. 4).

**Fig. 5:**
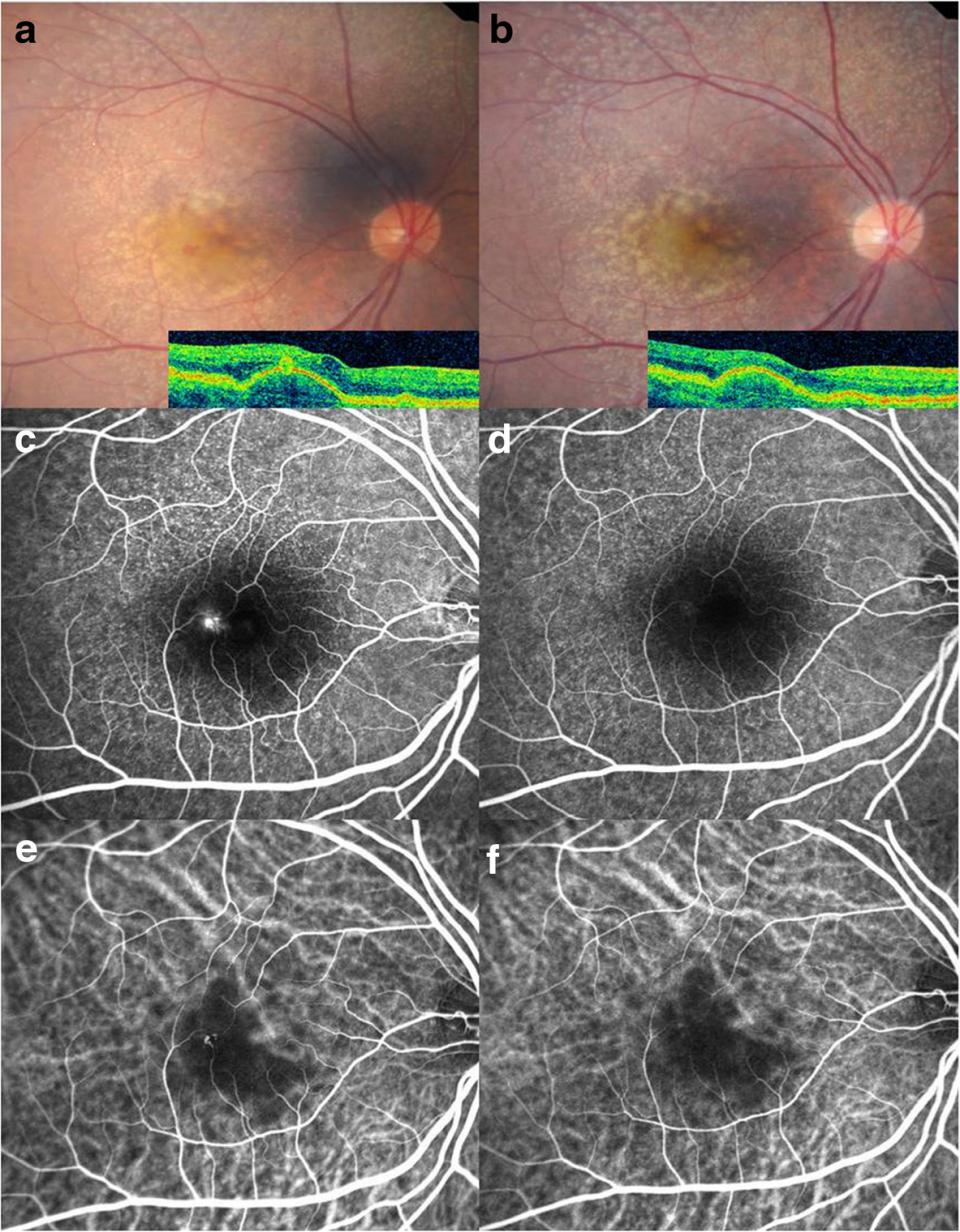
**a** Baseline fundus photograph and corresponding OCT image of a 62-year-old woman with retinal angiomatous proliferation (RAP) before injections. It demonstrates intraretinal neovascularization (IRN) with multiple drusen in the region of the fovea. The initial BCVA was 0.398 (LogMAR). **b** 12 months after injection, IRN decreased. The final BCVA was 0.201 (LogMAR). **c** FA shows a focal area of hyperfluorescence (leakage) corresponding to the IRN. **d** After injections, leakage in FA resolved in the IRN. **e** ICGA shows a focal area of intense hyperfluorescence or a so-called ‘hot spot’ corresponding to the lesion. **f** One year after treatment, ICGA showed resolution of hyperfluorescence

Seven eyes (87.5 %) in Group I did not show relapses during the follow-up periods (Fig. 5). RAP relapses were seen in 13 eyes (76.5 %), 12 eyes (87.5 %) in Group II and III, respectively. One eye (6.3 %) in Group III experienced small submacular hemorrhage after injections of ranibizumab. No eye in the three subgroups showed progression to a higher stage of RAP during the follow-up. No major ocular injection-related adverse events, including infectious endophthalmitis, drug-related inflammation, retinal detachment, or significantly increased intraocular pressure were observed during the follow-up period.

## Discussion

RAP accounts for 10 % to 15 % of all neovascular AMD in white patients, but its natural course is believed to be worse than that of typical exudative AMD. Viola et al [3] reported that six of nine patients with stage I and all of the patients with stage II progressed to visual loss within 6 months. Therefore, early recognition and proper management of RAP are essential. Several studies have reported many treatment procedures to establish the best strategy for treating RAP lesions. Among them, anti-VEGF therapy has been investigated in various modalities, with some treatment regimens involving PDT and triamcinolone [10, 21, 22].

Rouvas et al [23, 24] reported that patients treated with ranibizumab monotherapy, intravitreal triamcinolone plus PDT, or ranibizumab plus PDT resulted in stabilization of the disease. But patients treated with the simultaneous combination of PDT and either ranibizumab or triamcinolone experienced several negative effects. PDT may increase the risk of photooxidative damage in the retina through intraretinal activation of extravasated verteporfin in the perifoveal cystic space and increase the risk of a retinal pigment epithelium tear. Blockage of VEGF by the anti-VEGF agent may enhance and prolong the photothrombotic effects on the physiologic choroid caused by PDT, leading to severe choroidal ischemia [23]. Even though ranibizumab with combined PDT therapy can lead to improved visual function, therapy using this combination may be complicated by the higher incidence of RPE tears and atrophic change [25].

In our study, patients were scheduled to receive three consecutive doses of intravitreal ranibizumab. Thereafter, re-treatment with ranibizumab was performed based on defined criterion such as the one given in the injection schedule of PrONTO study [26]. Although the PrONTO study did not provide separate data for patients with RAP lesions, Fung et al [27] reported that patients with RAP required a higher mean number of injections of ranibizumab compared to patients with other types of CNV in the PrONTO study.

In our results, treatment prognosis differs according to the clinical stage of RAP during the 12 months. In several recent reports, intravitreal ranibizumab monotherapy was given mostly to patients with stage II and III RAP lesions [28, 29]. Parodi et al [30] demonstrated that both ranibizumab and bevacizumab showed similar efficacy in stage I and II RAP. But they did not analyse the subgroup within the ranibizumab injection group. Kramann et al [19] reported the short term results of intravitreal ranibizumab for 26 eyes with RAP including 7 eyes of stage I RAP. 12 eyes had 6 months follow-up, and 6 eyes completed 9-months follow-up. It would be difficult to evaluate the relapse of RAP with such short follow-up periods.

In our study, there was an improvement in visual acuity and CMT in all groups after the intravitreal ranibizumab injections. Although improvement of Group I showed similar trend of ‘ceiling effect’ observed in the ANCHOR study [31], BCVA and CMT in group I at 12 months follow-up showed the most improvement among three groups , resulting in the best functional and anatomical outcomes. In addition, lower number of reinjections needed in Group I means that intravitreal ranibizumab treatment is effective in preventing recurrence in patients with stage I RAP. Stage II and stage III lesions seem to be more difficult to treat, because theses lesions have PED or chorioretinal anastomoses. This may be one of the reasons why RAP is known to have a poorer prognosis than neovascular AMD. In the PrONTO study [26], RAP lesions received 5.8 injections per year, and the number of ranibizumab injections showed a higher frequency than other types of AMD. In comparison, the mean number of injections (4.07) in our study seems to be lower than that of the PrONTO study. But, except for stage I, RAP at stages II and III received 4.88 injections, which was a similar number to the one in the PrONTO study, although a direct comparison is not possible. Especially, patients in stage I RAP presented relatively few recurrences and responded well compared with patients in stages II and III. Therefore, these results showed that the stage of RAP could be an important prognostic factor.

With regard to the combination of an anti-VEGF agent with PDT for RAP, Lee et al [32] reported an improvement in the mean visual acuity from 20/125 at the baseline to 20/63 at the 12-month follow-up of 9 eyes treated with ranibizumab in conjunction with PDT in stages II and III. But the fact that geographic atrophy was detected in the groups having combined PDT with an anti-VEGF agent suggests that simultaneous combination treatment with PDT in patients with RAP may enhance photochemical stress in the normal choroid with prolonged or magnified hypofluorescence in ICGA because of ischemia in the normal choriocapillaries. Sometimes photodynamic therapy can lead to significant adverse effects such as massive hemorrhage, RPE damage and fibrous scar formation; such complications are a serious concern and cannot be overlooked.

Rouvas et al [33] reported that lesions that are believed to be Stage II may in reality be Stage III because of the presence of small undetectable chorioretinal anastomoses. We should understand the difference in prognosis according to the stage of the disease. In the present study, it is possible to say that because the mean number of injections was lower in stage I, patients in this group experienced a lower rate of complications and recurrences in comparison with patients in other stages. These findings have important implications in the prognosis of RAP in that the early diagnosis of stage I RAP is critical in preventing recurrence of RAP. Although no eye in the three subgroups showed progression to a higher stage of RAP during the follow-up, additional therapies such as more frequent ranibizumab injection and combined with PDT can be considered in stages II and III.

## Conclusions

Although our study has some limitations such as the retrospective nature of the study, the absence of a control group, a small number of patients, and a relatively short follow-up time, our results suggest that ranibizumab monotherapy may be a safe and highly effective treatment option for patients with RAP lesions that are in early stage. On the other hand, selective individuals in late stage are required more additional therapies. A larger, controlled, prospective, randomized comparative study with a longer follow-up period will be required to fully compare differences in treatment efficacy according to the clinical staging of RAP.

## Abbreviations

AMD: age-related macular degeneration
BCVA: best-corrected Snellen visual acuity
CMT: central macular thickness
CNV: choroidal neovascularization
FA: fluorescein angiography
ICGA: indocyanine green angiography
IRN: intraretinal neovascularization
OCT: optical coherence tomography
PDT: photodynamic therapy
PED: pigment epithelium detachment
RAP: retinal angiomatous proliferation
SRN: subretinal neovascularization

## References

[1] Yannuzzi LA, Negrao S, Iida T, Carvalho C, Rodriguez-Coleman H, Slakter J (2001). Retinal angiomatous proliferation in age-related macular degeneration. Retina.

[2] Freund KB, Ho IV, Barbazetto IA, Koizumi H, Laud K, Ferrara D (2008). Type 3 neovascularization: the expanded spectrum of retinal angiomatous proliferation. Retina.

[3] Viola F, Massacesi A, Orzalesi N, Ratiglia R, Staurenghi G (2009). Retinal angiomatous proliferation: natural history and progression of visual loss. Retina.

[4] Bressler NM (2005). Retinal anastomosis to choroidal neovascularization: a bum rap for a difficult disease. Arch Ophthalmol.

[5] Bottoni F, Massacesi A, Cigada M, Viola F, Musicco I, Staurenghi G (2005). Treatment of retinal angiomatous proliferation in age-related macular degeneration: a series of 104 cases of retinal angiomatous proliferation. Arch Ophthalmol.

[6] Kuroiwa S, Arai J, Gaun S, Iida T, Yoshimura N (2003). Rapidly progressive scar formation after transpupillary thermotherapy in retinal angiomatous proliferation. Retina.

[7] Sakimoto S, Gomi F, Sakaguchi H, Tano Y (2005). Recurrent retinal angiomatous proliferation after surgical ablation. Am J Ophthalmol.

[8] Boscia F, Furino C, Sborgia L, Reibaldi M, Sborgia C (2004). Photodynamic therapy for retinal angiomatous proliferations and pigment epithelium detachment. Am J Ophthalmol.

[9] Saito M, Iida T, Kano M (2012). Combined intravitreal ranibizumab and photodynamic therapy for retinal angiomatous proliferation. Am J Ophthalmol.

[10] Rouvas AA, Papakostas TD, Vavvas D, Vergados I, Moschos MM, Kotsolis A (2009). Intravitreal ranibizumab, intravitreal ranibizumab with PDT, and intravitreal triamcinolone with PDT for the treatment of retinal angiomatous proliferation: a prospective study. Retina.

[11] Kliffen M, Sharma HS, Mooy CM, Kerkvliet S, De Jong PT (1997). Increased expression of angiogenic growth factors in age-related maculopathy. Br J Ophthalmol.

[12] Oh H, Takagi H, Takagi C, Suzuma K, Otani A, Ishida K (1999). The potential angiogenic role of macrophages in the formation of choroidal neovascular membranes. Invest Ophthalmol Vis Sci.

[13] Rosenfeld PJ, Brown DM, Heier JS, Boyer DS, Kaiser PK, Chung CY (2006). Ranibizumab for neovascular age-related macular degeneration. N Engl J Med.

[14] Brown DM, Kaiser PK, Michels M, Soubrane G, Heier JS, Kim RY (2006). Ranibizumab versus verteporfin for neovascular age-related macular degeneration. N Engl J Med.

[15] Meyerle CB, Freund KB, Iturralde D, Spaide RF, Sorenson JA, Slakter JS (2007). Intravitreal bevacizumab (Avastin) for retinal angiomatous proliferation. Retina.

[16] Shimada H, Kawamura A, Mori R, Yuzawa M (2007). Clinicopathological findings of retinal angiomatous proliferation. Graefes Arch Clin Exp Ophthalmol.

[17] Rouvas A, Petrou P, Vergados I, Pechtasides D, Liarakos V, Mitsopoulou M (2009). Intravitreal ranibizumab (Lucentis) for treatment of central retinal vein occlusion: a prospective study. Graefes Arch Clin Exp Ophthalmol.

[18] Montero JA, Fernandez MI, Gomez-Ulla F, Ruiz-Moreno JM (2009). Efficacy of intravitreal bevacizumab to treat retinal angiomatous proliferation stage II and III. Eur J Ophthalmol.

[19] Kramann CA, Schopfer K, Lorenz K, Zwiener I, Stoffelns BM, Pfeiffer N (2012). Intravitreal ranibizumab treatment of retinal angiomatous proliferation. Acta Ophthalmol.

[20] Atmani K, Voigt M, Le Tien V, Querques G, Coscas G, Soubrane G (2010). Ranibizumab for retinal angiomatous proliferation in age-related macular degeneration. Eye (Lond).

[21] Nakano S, Honda S, Oh H, Kita M, Negi A (2012). Effect of photodynamic therapy (PDT), posterior subtenon injection of triamcinolone acetonide with PDT, and intravitreal injection of ranibizumab with PDT for retinal angiomatous proliferation. Clin Ophthalmol.

[22] Sahu AK, Narayanan R (2010). Intravitreal ranibizumab, intravitreal ranibizumab with photodynamic therapy (PDT), and intravitreal triamcinolone with PDT for the treatment of retinal angiomatous proliferation. Retina.

[23] Rouvas AA, Papakostas TD, Ladas ID, Vergados I (2008). Enlargement of the hypofluorescent post photodynamic therapy treatment spot after a combination of photodynamic therapy with an intravitreal injection of bevacizumab for retinal angiomatous proliferation. Graefes Arch Clin Exp Ophthalmol.

[24] Rouvas AA, Chatziralli IP, Theodossiadis PG, Moschos MM, Kotsolis AI, Ladas ID (2012). Long-term results of intravitreal ranibizumab, intravitreal ranibizumab with photodynamic therapy, and intravitreal triamcinolone with photodynamic therapy for the treatment of retinal angiomatous proliferation. Retina.

[25] McBain VA, Kumari R, Townend J, Lois N (2011). Geographic atrophy in retinal angiomatous proliferation. Retina.

[26] Lalwani GA, Rosenfeld PJ, Fung AE, Dubovy SR, Michels S, Feuer W (2009). A variable-dosing regimen with intravitreal ranibizumab for neovascular age-related macular degeneration: year 2 of the PrONTO Study. Am J Ophthalmol.

[27] Fung AE, Lalwani GA, Rosenfeld PJ, Dubovy SR, Michels S, Feuer WJ (2007). An optical coherence tomography-guided, variable dosing regimen with intravitreal ranibizumab (Lucentis) for neovascular age-related macular degeneration. Am J Ophthalmol.

[28] Konstantinidis L, Mameletzi E, Mantel I, Pournaras JA, Zografos L, Ambresin A (2009). Intravitreal ranibizumab (Lucentis) in the treatment of retinal angiomatous proliferation (RAP). Graefes Arch Clin Exp Ophthalmol.

[29] Maier M, Perz C, Bockmaier J, Feucht N, Lohmann CP (2012). Therapy of stage III retinal angiomatous proliferation : Intravitreal ranibizumab injections. Ophthalmologe.

[30] Parodi MB, Iacono P, Menchini F, Sheth S, Polini G, Pittino R, et al. Intravitreal bevacizumab versus ranibizumab for the treatment of retinal angiomatous proliferation. Acta Ophthalmol. 2011. 10.1111/j.1755-3768.2011.02265.x 21951313

[31] Kaiser PK, Brown DM, Zhang K, Hudson HL, Holz FG, Shapiro H (2007). Ranibizumab for predominantly classic neovascular age-related macular degeneration: subgroup analysis of first-year ANCHOR results. Am J Ophthalmol.

[32] Lee MY, Kim KS, Lee WK (2011). Combination therapy of ranibizumab and photodynamic therapy for retinal angiomatous proliferation with serous pigment epithelial detachment in Korean patients: twelve-month results. Retina.

[33] Rouvas AA, Papakostas TD, Ntouraki A, Douvali M, Vergados I, Ladas ID (2010). Angiographic and OCT features of retinal angiomatous proliferation. Eye (Lond).

